# CARD8 regulates basal inflammatory signaling and UPEC colonization in bladder epithelial cells

**DOI:** 10.1186/s10020-026-01686-z

**Published:** 2026-10-31

**Authors:** Geena V. Paramel, Isak Demirel

**Affiliations:** School of Medical Sciences, Faculty of Medicine and Health, Örebro University, Örebro, SE-701 82 Sweden

**Keywords:** UPEC, Urinary tract infection, CARD8, Invasion, Bladder epithelial cells

## Abstract

**Background:**

Urinary tract infection (UTI) is commonly caused by uropathogenic *Escherichia coli* (UPEC), which can modulate epithelial inflammatory responses and invade bladder epithelial cells. Inflammasome-related pathways are important during UPEC infection, but the role of the inflammasome-associated protein CARD8 in bladder epithelial cells remains unknown. The aim of this study was to investigate the role of CARD8 in bladder epithelial cell responses to UPEC infection.

**Method:**

Human bladder epithelial cells (HBLAK) were transfected with CARD8 siRNA or control siRNA and infected with the UPEC strain CFT073. Inflammatory responses were assessed by RT-qPCR, ELISA, LDH release, caspase-1 activity assay, and western blotting. Bacterial colonization and invasion were analyzed using fluorescence-based and gentamicin protection assays. To evaluate the role of major UPEC virulence factors, cells were infected with CFT073 mutants lacking fimH, pap or hlyA.

**Results:**

CARD8 knockdown reduced basal IL-1β and NLRP3 mRNA expression, pro-IL-1β and caspase-1 protein levels, and basal IL-1β and IL-8 release. UPEC infection selectively downregulated the 47-kDa CARD8 isoform, and this effect was not dependent on FimH, Pap, TcpC or HlyA. In addition, CARD8 knockdown did not attenuate CFT073-induced IL-1β release, LDH release, or caspase-1 activity. Furthermore, CARD8 knockdown increased CFT073 colonization and invasion and was associated with increased UPK1A mRNA expression.

**Conclusion:**

This study identifies CARD8 as a novel regulator of bladder epithelial responses to UPEC infection. CARD8 contributes to basal inflammatory signaling and limits bacterial colonization and invasion. This suggests that CARD8 may regulate epithelial immune responses and host-pathogen interactions during UTI.

## Background

Urinary tract infection (UTI) is one of the most common bacterial infections worldwide and represents a major clinical burden, particularly in women, where recurrent infections are frequent (Flores-Mireles et al. 2015, Foxman 2010). The predominant cause of UTI is uropathogenic *Escherichia coli* (UPEC), which has evolved several virulence strategies that enable colonization, persistence, immune modulation and tissue damage within the urinary tract (Flores-Mireles et al. 2015, Foxman 2010, Bien et al. 2012, Wiles et al. 2008). Successful UPEC infection depends on the coordinated expression of multiple virulence factors, including type-1 fimbriae, P-fimbriae, α-hemolysin, lipopolysaccharide, siderophores, and other immune-modulatory factors (Bien et al. 2012, Wiles et al. 2008). Type-1 fimbriae mediate attachment to the bladder epithelium and promote invasion of urothelial cells, while P-fimbriae is associated with upper urinary tract colonization (Flores-Mireles et al. 2015, Bien et al. 2012, Wiles et al. 2008, Demirel et al. 2018). In addition, α-hemolysin is an important pore-forming toxin that can modulate epithelial cell signaling, promote exfoliation and contribute to tissue damage (Bien et al. 2012, Wiles et al. 2008, Demirel et al. 2018, Mulvey et al. 2001, Dhakal and Mulvey 2012, Nagamatsu et al. 2015, Ristow and Welch 2016). UPEC can also invade bladder epithelial cells and form intracellular bacterial communities and quiescent reservoirs, which may protect the bacteria from immune clearance and antibiotic treatment and thereby contribute to recurrent infection (Mulvey et al. 2001, Hannan et al. 2012, Rosen et al. 2007).

The urinary tract epithelium is not only a physical barrier but also an active immunological barrier that detects invading pathogens and initiates inflammatory responses. Upon UPEC infection, bladder epithelial cells produce cytokines and chemokines such as IL-6, IL-8, and IL-1β, which contribute to immune cell recruitment and activation (Demirel et al. 2018, Billips et al. 2008, Hunstad et al. 2005, Kuhn et al. 2023). Among these mediators, IL-1β is of particular interest because it is generated as an inactive precursor that requires inflammasome-mediated processing by caspase-1 before secretion (Demirel et al. 2018, Nagamatsu et al. 2015, Kuhn et al. 2023, Ambite et al. 2016). Inflammasomes are cytosolic multiprotein complexes that sense microbial and danger-associated signals and regulate caspase-1 activation, IL-1β and IL-18 maturation, and inflammatory cell death (Yao et al. 2024). The NLRP3 inflammasome has been implicated in the pathogenesis and outcome of UTI, and previous studies have shown that UPEC strain CFT073 can activate caspase-1 and induce IL-1β release from bladder epithelial cells in a virulence factor-dependent manner (Demirel et al. 2018, Nagamatsu et al. 2015, Ambite et al. 2016). In particular, α-hemolysin has been shown to regulate NLRP3 inflammasome activation and IL-1β release, while type 1 fimbriae can influence bacterial colonization and invasion of bladder epithelial cells (Demirel et al. 2018, Nagamatsu et al. 2015).

CARD8 is an inflammasome-related protein that has emerged as an important but cell type- and stimulus-dependent regulator of inflammation. Earlier studies described CARD8 mainly as an anti-inflammatory regulator capable of suppressing NF-κB signaling, NOD2-dependent responses, and NLRP3-associated caspase-1 activation (Bouchier-Hayes et al. 2001, Razmara et al. 2002, Kampen et al. 2010). More recent work has shown that CARD8 can also function as an inflammasome sensor (D’Osualdo et al. 2011, Sharif et al. 2021). CARD8 contains a FIIND region and a C-terminal CARD domain, and following FIIND autoproteolysis and degradation of the inhibitory N-terminal region, the C-terminal fragment can directly recruit caspase-1 and promote pyroptotic signaling (D’Osualdo et al. 2011, Sharif et al. 2021). These apparently divergent functions suggest that CARD8 activity depends strongly on cell type, isoform expression, and inflammatory context (Bouchier-Hayes et al. 2001, Razmara et al. 2002, Kampen et al. 2010, D’Osualdo et al. 2011, Sharif et al. 2021, Ito et al. 2014). The complexity of CARD8 biology is further illustrated by the C10X polymorphism, which has been associated with several inflammatory and infectious diseases, although its functional consequences remain incompletely understood (Abate et al. 2019, Asfaw Idosa et al. 2014, Belayneh et al. 2024, Kastbom et al. 2010, Sangal et al. 2021). While C10X is generally considered a loss-of-function variant, alternative CARD8 transcripts, including the 47-kDa T47 isoform, initiate downstream of the premature stop codon and may therefore escape this mutation(Asfaw Idosa et al. 2014). This suggests that residual CARD8 activity may vary between cell types and inflammatory settings, highlighting the need for experimental models that directly define CARD8 function and isoform regulation during bacterial infection. The role of CARD8 in barrier tissues is still incompletely understood. In endothelial cells, CARD8 is functionally important and has been linked to regulation of inflammatory mediators such as IL-6 and IL-8 (Paramel et al. 2020). In epithelial cells, CARD8 has been reported to negatively regulate NOD2-dependent inflammatory signaling in intestinal epithelial cells (Kampen et al. 2010), while CARD8 inflammasome signaling can activate caspase-1-dependent pathways in keratinocytes (Gong et al. 2021). However, despite the established importance of inflammasome signaling during UPEC infection, the role of CARD8 in bladder epithelial responses to UPEC has not been investigated to our knowledge. This represents an important knowledge gap, particularly because UPEC-induced inflammation involves caspase-1 activation, IL-1β release, IL-8 production, cytotoxicity, and bacterial colonization, all of which may potentially be influenced by CARD8-dependent signaling. The aim of this study was therefore to investigate the role of CARD8 in bladder epithelial cell responses to UPEC infection.

## Materials and methods

### Cell culture

The spontaneously immortalized human bladder epithelial cells HBLAK (CELLnTEC, Advanced Cell Systems AG, Bern, Switzerland) and the primary bladder epithelial cell line HBEP were cultured in Epilife medium with 60 µM calcium chloride supplemented with Human Keratinocyte Growth Supplement (HKGS, Thermo Fisher Scientific, Waltham, MA, United States) at 37 °C with 5% CO_2_. At confluency, HBLAK and HBEP were differentiated into umbrella-like cells for 24 h in EpiLife medium supplemented with HKGS and 1 mM CaCl₂, and the same medium was used during infection experiments. The human bladder epithelial cell line 5637 (ATCC HTB-9) was obtained from the American Type Culture Collection (Manassas, VA, USA). Cells were maintained in Dulbecco’s Modified Eagle Medium (DMEM; BioWhittaker, Lonza, Basel, Switzerland) supplemented with 2 mM L-glutamine (Hyclone, GE Life Sciences, UK), 10% fetal bovine serum (FBS), and 1 mM non-essential amino acids at 37 °C in a humidified atmosphere containing 5% CO₂. During experimental procedures, the FBS concentration was reduced from 10% to 2%.

### Bacterial strains

The UPEC strain CFT073 was originally isolated from a patient with pyelonephritis (Mobley et al. 1990), and the corresponding CFT073 deletion mutants ΔfimH, Δpap, ΔTcpC and ΔhlyA were used in this study (Demirel et al. 2018). The CFT073Δpap mutant was kindly provided by Professor Harry Mobley, University of Michigan Medical School, USA. The CFT073ΔTcpC mutant was kindly provided by Professor Catharina Svanborg, Lund University, Sweden. The clinical UPEC isolate was obtained from the Department of Microbiology at Örebro University hospital, Sweden, from an anonymized individual with suspected pyelonephritis. The cystitis strain UTI89 and the MG1655, a nonpathogenic *E. coli* K-12 strain, were also used. Bacteria were maintained on tryptic soy agar and cultured overnight in Luria broth (Lennox; Franklin Lakes, NJ, USA) at 37 °C under aerobic conditions with shaking. Prior to infection experiments, bacteria were harvested and resuspended in phosphate-buffered saline (PBS) to the appropriate concentrations.

### Transfection of HBLAK using siRNA

The HBLAK cells were plated in 24-well (150000 cells/well) or 96-well (30000 cells/well) plates and allowed to grow overnight and transfected with siRNA followed by incubation for 24 h. Cells were transfected with a pool of three CARD8 stealth RNAi (Cat. no. 53621349, Stealth siRNA CARD8: HSS117857, HSS117858, HSS177013; Thermo Fisher Scientific) or stealth RNAi siRNA Negative Control Med GC Duplex (Cat. no. 462001, Thermo Fisher Scientific) at a final concentration of 10 nM, with Lipofectamine 2000 (Cat. no. 11668-019, Thermo Fisher Scientific), diluted in Epilife medium with 60 µM calcium chloride supplemented with HKGS, according to the manufacturer’s protocol. Following transfection, cells were differentiated for 24 h prior to infection.

### Cell infection procedures

The HBLAK cells were infected with the UPEC strain CFT073 and the corresponding CFT073 deletion mutants ΔfimH, Δpap, ΔTcpC and ΔhlyA. The HBEP and the 5637 cells were infected with CFT073 for 5 h. Prior to infection, HBLAK cells were transfected with CARD8 siRNA or negative control siRNA and subsequently differentiated for 24 h into umbrella-like cells. Differentiated HBLAK, HBEP and 5637 cells were infected at a multiplicity of infection (MOI) of 1 or 10 and incubated for 3–5 h at 37 °C with 5% CO₂. Following infection, supernatants, RNA, and/or protein lysates were collected, depending on downstream analysis, and stored at − 80 °C until further use. The MOI was selected according to each assay. MOI 1 and 10 were used for inflammatory experiments to limit bacterial burden and cytotoxicity, while higher MOIs were required for colonization and invasion because only a small proportion of bacteria adhere to or enter epithelial cells during the short infection period.

### RNA isolation, cDNA synthesis and quantitative polymerase chain reaction

Total RNA was extracted using the E.Z.N.A.^®^ Total RNA Kit I (Omega Bio-tek, Norcross, GA, USA) in accordance with the manufacturer’s protocol. RNA yield and purity were determined using a NanoDrop 2000 spectrophotometer (Thermo Fisher Scientific). Complementary DNA (cDNA) was synthesized from 100 ng total RNA using the High-Capacity cDNA Reverse Transcription Kit (Thermo Fisher Scientific). Quantitative real-time PCR (RT-qPCR) was performed using 5 ng cDNA, 250 nM of each primer (Table 1; Eurofins MWG Synthesis GmbH, Ebersberg, Germany), and Maxima SYBR Green qPCR Master Mix (Thermo Fisher Scientific). Reactions were run on a QuantStudio 7 Flex real-time PCR system (Thermo Fisher Scientific) for 40 cycles consisting of denaturation at 95 °C for 15 s, annealing at 60 °C for 30 s, and extension at 72 °C for 30 s. Following amplification, melting curve analysis was performed from 60 °C to 95 °C to verify amplification specificity. Cycle threshold (Ct) values were recorded, and relative gene expression was calculated using the ΔΔCt method (2^⁻ΔΔCt^), with GAPDH used as the endogenous reference gene.

**Table 1:** Primers used for quantitative real time PCR

Gene symbol	Oligonucleotide sequences (5′–3′)
*IL-1β*	F: CCACAGACCTTCCAGGAGAATG R: GTGCAGTTCAGTGATCGTACAGG
*IL-8*	F: GAGAGTGATTGAGAGTGGACCAC R: CACAACCCTCTGCACCCAGTTT
*Caspase-1*	F: GCTGAGGTTGACATCACAGGCA R: TGCTGTCAGAGGTCTTGTGCTC
*NLRP3*	F: GGACTGAAGCACCTGTTGTGCA R: TCCTGAGTCTCCCAAGGCATTC
*CARD8*	F: GTGTGGGATACTGAGGTGAAGC R: TGTCCTGGAGATCATCGAGCAC
*UPK1A*	F: CACCAAGCAGATGCTGACCTTC R: GGACCAGATGTGCCACAGCATT
*UPK3A*	F: CTCACAGATCCTGAATGCCTACC R: CCGTGGACATATTGACCAGGAC
*ITGA3*	F: GCCTGACAACAAGTGTGAGAGC R: GGTGTTCGTCACGTTGATGCTC
*ITGB1*	F: GGATTCTCCAGAAGGTGGTTTCG R: TGCCACCAAGTTTCCCATCTCC
*GAPDH*	F: GTCTCCTCTGACTTCAACAGCG R: ACCACCCTGTTGCTGTAGCCAA

### Western blot

Total protein was extracted using ice-cold 1× RIPA lysis buffer supplemented with a protease inhibitor cocktail (Thermo Fisher Scientific). Protein concentration was quantified using the BCA Protein Assay Kit (Thermo Fisher Scientific) according to the manufacturer’s instructions. Equal amounts of protein (8–10 µg) were denatured in 4×SDS sample buffer at 95 °C for 5 min and separated by SDS-PAGE using 8–16% Criterion™ TGX Stain-Free™ Precast Gels (Bio-Rad, Hercules, CA, USA) with 10×Tris/Glycine/SDS running buffer. Proteins were transferred onto nitrocellulose membranes using the Trans-Blot^®^ Electrophoretic Transfer Cell (Bio-Rad) and 10×Tris/Glycine transfer buffer. After blocking in 5% non-fat dry milk in TBS-T (10 mM Tris-HCl pH 8.0, 150 mM NaCl, 0.1% Tween-20) for 45 min, membranes were incubated overnight at 4 °C with the following primary antibodies: phospho-P38 (4511 S, 1:1000, Cell Signaling Technology, Danvers, MA, USA), Total-P38 (9212 S, 1:1000, Cell Signaling Technology), CARD8 (PAB0281, 1:1000, Abnova, Taipei City, Taiwan), NLRP3 (MAB3724, 1:1000, Abnova, Taipei City, Taiwan), IL-1β (#GTX100793, 1:1000, GeneTex, Hinschu City, Taiwan), Caspase-1 AG-20B-0048, 1:1000, Adipogen, COGER SAS, Paris, France), and GAPDH (FL-335, 1:5000; Santa Cruz Biotechnology, Dallas, TX, USA). After washing, membranes were incubated for 2 h at room temperature with HRP-conjugated anti-rabbit or anti-mouse secondary antibodies (1:2000). Protein bands were visualized using a Western-Ready™ ECL Substrate Premium Kit (BioLegend, San Diego, CA, USA), and chemiluminescence was detected using the ChemiDoc™ MP Imaging System (Bio-Rad). Densitometric analysis was conducted using Image Lab 5.0 software (Bio-Rad).

### Cytokine release and viability assay

Supernatants were collected after bacterial infection of HBLAK cells and centrifuged for 5 min at 5,000 g and stored at − 80 °C. Enzyme-linked immunosorbent assays (ELISAs) were performed to quantify the release of interleukin-1β (IL-1β), IL-6 and IL-8 from HBLAK using commercially available kits (ELISA MAX Deluxe Sets, BioLegend, San Diego, CA, USA), according to the manufacturer’s instructions. Cell viability was also evaluated using the CyQUANT™ LDH Cytotoxicity Assays (Thermo Fisher Scientific), according to the manufacturer’s instructions.

### Caspase-1 activity assay

HBLAK cells were plated in a 96-well plate and transfected as described above. Prior to infection, cells were pre-incubated with the caspase-1 substrate Ac-YVAD-AMC (Enzo Life Sciences, New York, NY, USA) for 1 h at 37 °C with 5% CO₂. Cells were then infected with CFT073 at an MOI 1, and caspase-1 activity was measured after 3 and 5 h using a fluorescent plate reader at excitation/emission wavelengths of 340/440 nm (Cytation 3, BioTek Inc., Winooski, VT, USA).

### Colonization and invasion assay

HBLAK cells were infected with CFT073 (eGFP, enhanced green fluorescence protein), at MOI 100 at 37 °C with 5% CO_2_ for 3 h to measure colonization (adhered/invaded bacteria). The wells were then washed 10 times with PBS and CFT073 (eGFP) were quantified and imaged with a Cytation 3 plate reader. Intracellular presence of CFT073 was assessed by infecting HBLAK cells with CFT073 at MOI 10 for 2 h at 37 °C with 5% CO_2_. The wells were washed 10 times with PBS after infection and the medium was replaced with Epilife medium supplemented with 100 µg/mL gentamicin and incubated for additional 2 h. The cells were thereafter washed three times with PBS and lysed with 0.1% Triton-x 100 in PBS (supplemented with calcium chloride 100 mg/l, and magnesium chloride 100 mg/l). The intracellular CFT073 were plated on TSA plates, incubated at 37 °C overnight and CFU was counted (Demirel et al. 2018).

### Statistics

All data was analysed with GraphPad Prism 9.0. Statistical differences between groups were analysed using Student’s unpaired *t*-test or one-way ANOVA followed by Bonferroni multiple testing correction. A *p*-value of < 0.05 was considered statistically significant. *n* represents the number of independent biological experiments. Results are presented as mean ± standard error of the mean (SEM).

## Results

### UPEC downregulates CARD8

**Fig. 1:**
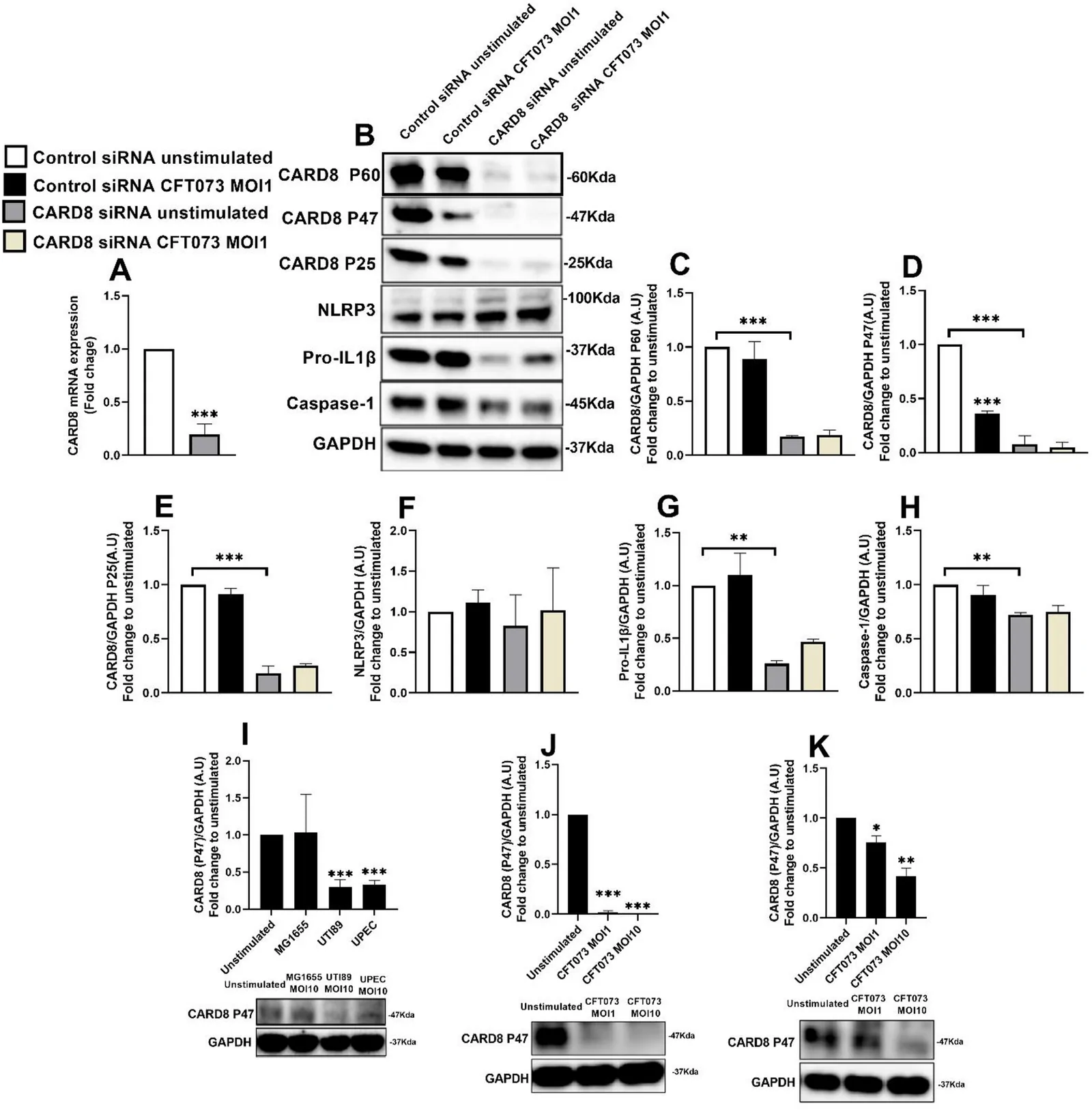
CFT073 downregulates the 47-kDa CARD8 isoform. CARD8 mRNA expression in HBLAK cells transfected with control siRNA or CARD8 siRNA after 24 h (**A**). HBLAK cells transfected with control siRNA or CARD8 siRNA and infected with CFT073 at MOI 1 for 5 h (**B-H**). Representative western blot of CARD8 isoforms, NLRP3, pro-IL-1β, caspase-1, and GAPDH (**B**). Densitometric analysis of the 60-kDa CARD8 isoform (C), 47-kDa CARD8 isoform (**D**), 25-kDa CARD8 isoform (**E)**, NLRP3 **(F)**, pro-IL-1β (G), and caspase-1 (**H**). 47-kDa CARD8 protein expression in HBLAK cells following infection with non-pathogenic *E. coli* MG1655, UTI89, or the clinical UPEC isolate (UPEC) at MOI 10 (**I)**. 47-kDa CARD8 protein expression in primary human bladder epithelial cells (HBEP) following CFT073 infection at MOI 1 or MOI 10 (**J**), and in 5637 bladder epithelial cells following CFT073 infection at MOI 1 or MOI 10 (**K**). Representative western blots and corresponding densitometric analyses are shown for panels I–K. Data are presented as mean ± SEM of *n* = 3 independent biological experiments. Asterisks indicate statistical significance compared to respective unstimulated: *p < 0.05, **p < 0.01, ***p < 0.001

To investigate the role of CARD8 during UPEC infection, HBLAK bladder epithelial cells were transfected with CARD8 siRNA or negative control siRNA and subsequently infected with CFT073. CARD8 silencing was confirmed at both the mRNA level (Fig. 1A) and protein level (Fig. 1B–E), showing a significant reduction in CARD8 P60, P47 and P25 isoform expression compared with unstimulated control-transfected cells. CARD8 knockdown also significantly reduced the protein levels of pro-IL-1β (Fig. 1B, G) and caspase-1 (Fig. 1B, H), whereas NLRP3 expression was not significantly affected (Fig. 1B, F). Notably, CFT073 infection of control-transfected cells significantly reduced the 47-kDa CARD8 isoform compared with unstimulated cells after 5 h (Fig. 1B, D), while no significant changes were observed for the 60-kDa or 25-kDa CARD8 isoforms (Fig. 1B, C, E). We also showed that the UPEC strain UTI89 and a clinical UPEC isolate, but not the commensal *E. coli* MG1655, significantly reduced the 47-kDa CARD8 isoform in HBLAK cells compared to unstimulated cells after 5 h (Fig. 1I). In addition, we also showed that CFT073 significantly reduced the 47-kDa CARD8 isoform in primary bladder epithelial cells (Fig. 1J) and in the bladder epithelial carcinoma cell line 5637 compared to unstimulated cells after 5 h (Fig. 1K). Collectively, these findings suggest that CARD8 contributes to the basal regulation of key inflammasome-related proteins in bladder epithelial cells and that UPEC infection specifically downregulates the 47-kDa CARD8 isoform.

### CARD8 regulates basal IL-1β and LDH release

**Fig. 2:**
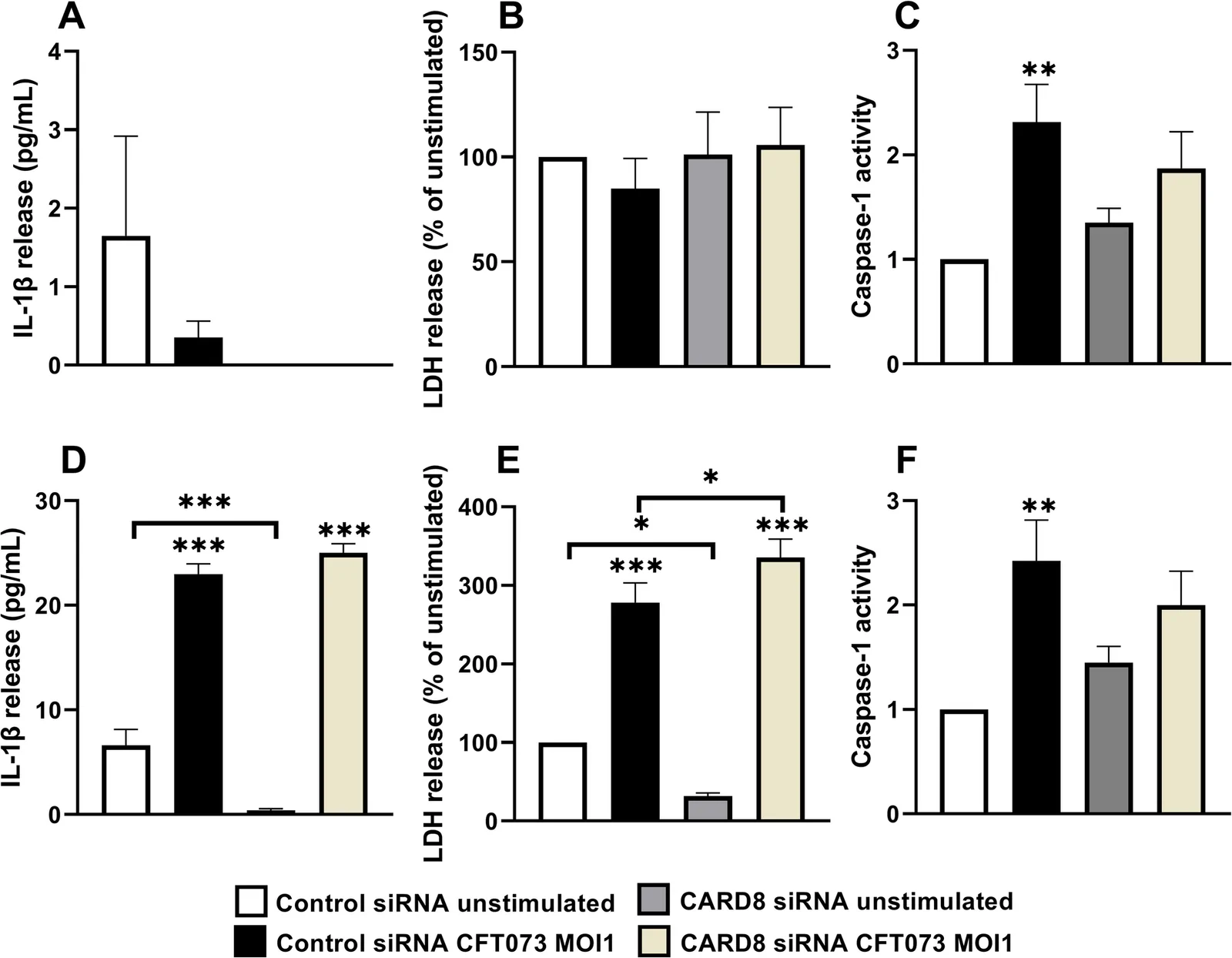
CARD8 regulates basal IL-1β and LDH release from bladder epithelial cells. Release of IL-1β (**A**), LDH release (**B**), and caspase-1 activity (**C**) from HBLAK cells transfected with control siRNA or CARD8 siRNA and infected with CFT073 at MOI 1 for 3 h (A-C) or 5 h (**D-F**). Data are presented as mean ± SEM of *n* = 5 independent biological experiments. Asterisks indicate statistical significance compared to respective unstimulated: *p < 0.05, **p < 0.01, ***p < 0.001

We next investigated whether CARD8 regulates IL-1β release, LDH release, and caspase-1 activity under basal conditions and following CFT073 infection. At 3 h, CARD8 knockdown did not significantly affect basal IL-1β release, LDH release, or caspase-1 activity compared with unstimulated control-transfected cells (Fig. 2A–C). However, CFT073 infection significantly increased caspase-1 activity in control-transfected cells at 3 h, whereas no significant increase in IL-1β or LDH release was observed (Fig. 2A–C). At 5 h, CARD8 knockdown significantly reduced basal IL-1β release and LDH release compared with unstimulated control-transfected cells, while basal caspase-1 activity was not significantly affected (Fig. 2D–F). CFT073 infection induced a significant increase in IL-1β release, LDH release, and caspase-1 activity in control-transfected cells at 5 h (Fig. 2D–F). However, these CFT073-induced responses were not significantly attenuated by CARD8 knockdown (Fig. 2D–F).

### CARD8 regulates basal IL-8 release in bladder epithelial cells

**Fig. 3:**
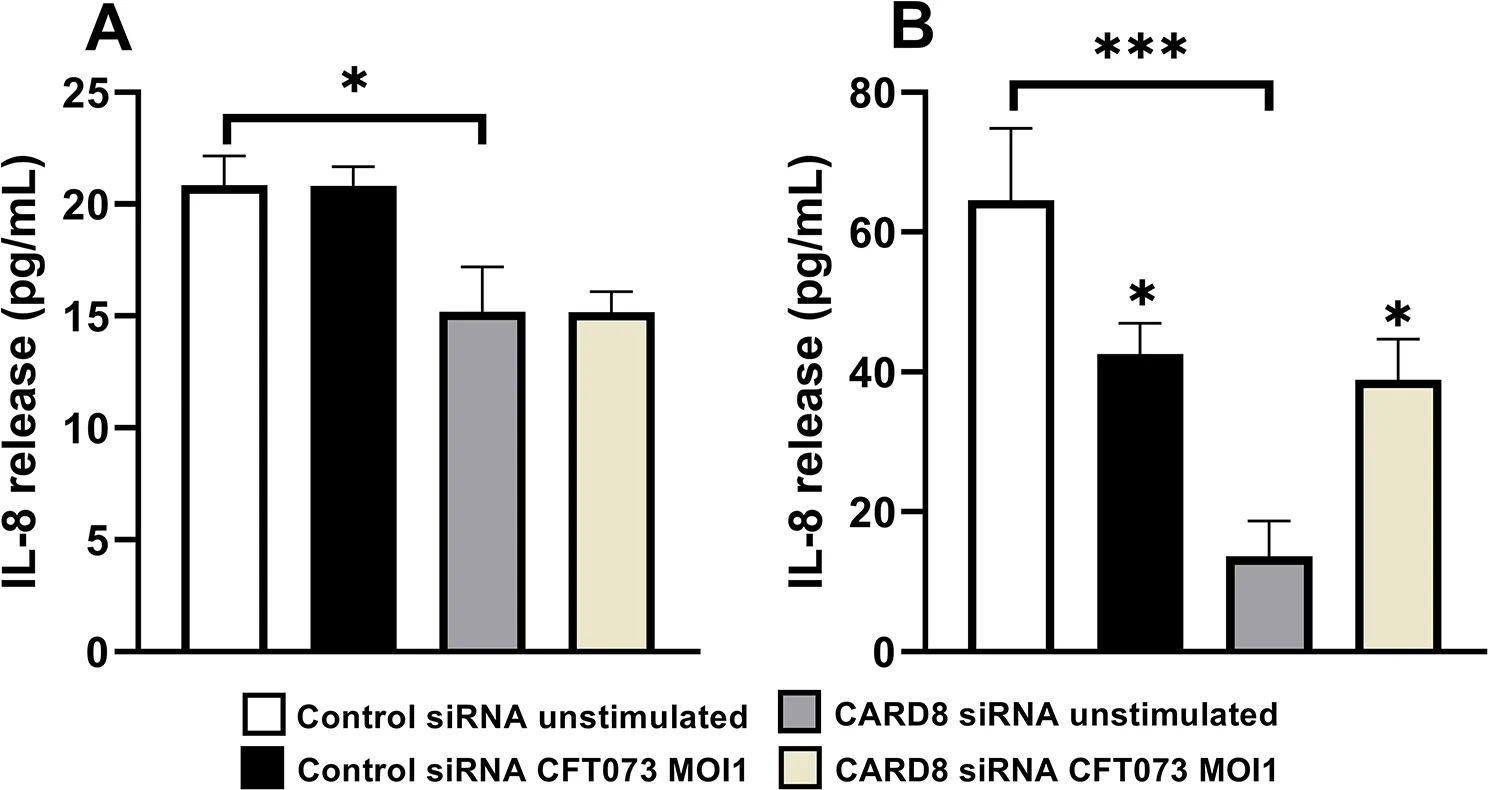
CARD8 regulates basal IL-8 release from bladder epithelial cells. Release of IL-8 from HBLAK cells transfected with control siRNA or CARD8 siRNA and infected with CFT073 at MOI 1 for 3 h (**A**) or 5 h (**B**). Data are presented as mean ± SEM of *n* = 4 independent biological experiments. Asterisks indicate statistical significance compared to respective unstimulated: *p < 0.05, ***p < 0.001

We next examined whether CARD8 regulates IL-8 release under basal conditions and following CFT073 infection. At 3 h, CARD8 knockdown significantly reduced IL-8 release compared with unstimulated control-transfected cells (Fig. 3A). At 5 h, CARD8 knockdown significantly reduced basal IL-8 release compared with unstimulated control-transfected cells (Fig. 3B). CFT073 infection significantly reduced IL-8 release in control-transfected cells compared with unstimulated control-transfected cells after 5 h. Furthermore, CFT073 infection of CARD8 knockdown cells increased IL-8 release compared with unstimulated CARD8 siRNA-transfected cells (Fig. 3B). We also assessed IL-6 release, but IL-6 was not detectable under these experimental conditions.

### CARD8 regulates basal IL-1β and NLRP3 gene expression

**Fig. 4:**
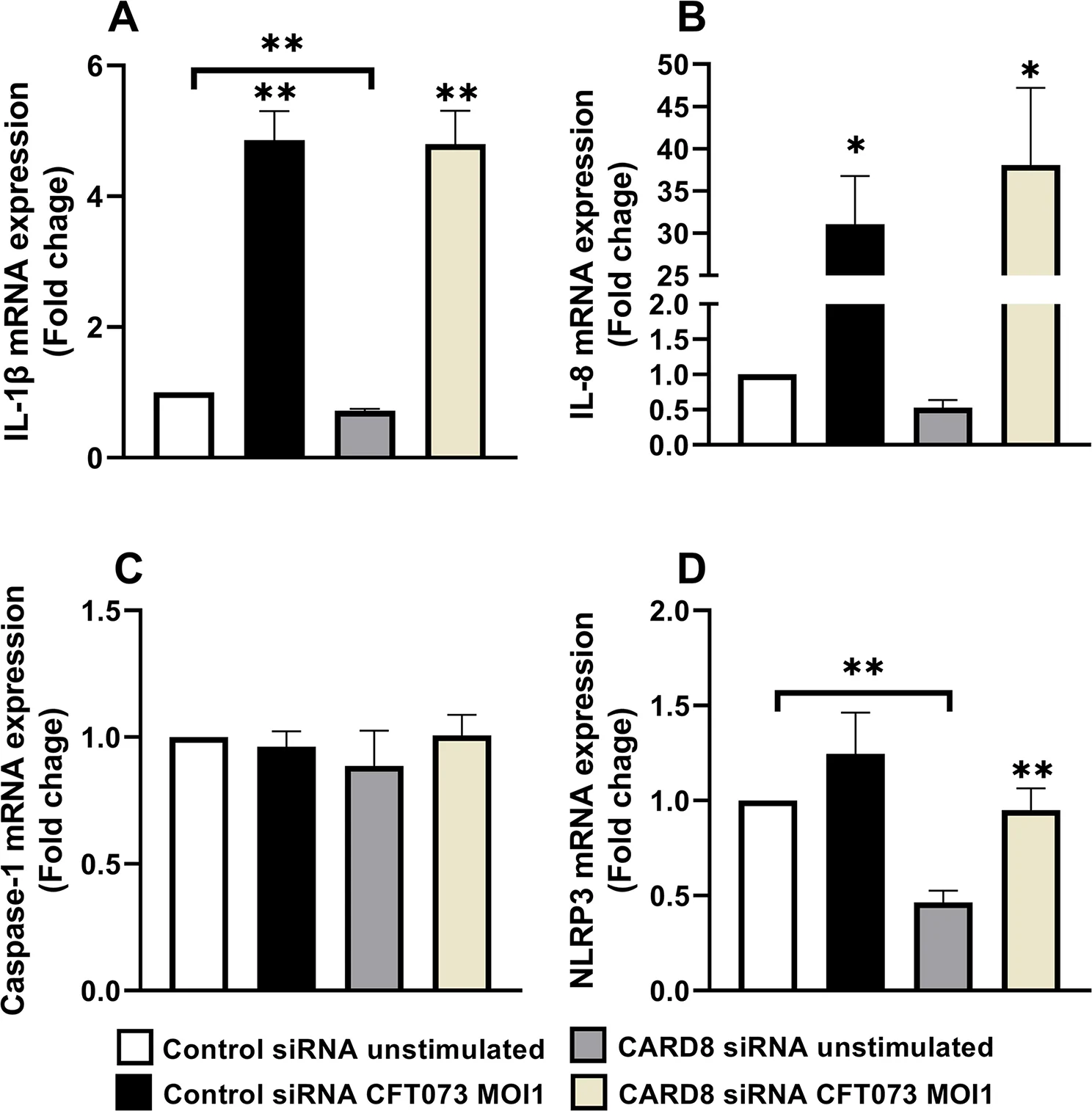
CARD8 regulates basal IL-1β and NLRP3 gene expression in bladder epithelial cells. Real-time qPCR analysis of IL-1β (**A**), IL-8 (**B**), caspase-1 (**C**), and NLRP3 (**D**) mRNA expression in HBLAK cells transfected with control siRNA or CARD8 siRNA and infected with CFT073 at MOI 1 for 5 h. Gene expression is presented as fold change relative to unstimulated control siRNA-transfected cells. Data are presented as mean ± SEM of *n* = 4 independent biological experiments. Asterisks indicate statistical significance compared to respective unstimulated: *p < 0.05, **p < 0.01

We next investigated whether CARD8 regulates the mRNA expression of inflammasome-related genes and IL-8 under basal conditions and following CFT073 infection. CARD8 knockdown significantly reduced basal IL-1β (Fig. 4A) and NLRP3 (Fig. 4D) mRNA expression compared with unstimulated control-transfected cells, whereas caspase-1 (Fig. 4C) and IL-8 (Fig. 4B) mRNA expression were not significantly affected. Following CFT073 infection, IL-1β and IL-8 mRNA expression were significantly increased in both control-transfected and CARD8-silenced cells (Fig. 4A, B), indicating that CARD8 knockdown did not attenuate the CFT073-induced upregulation of these genes. In contrast, caspase-1 mRNA expression remained unchanged following CFT073 infection in both control-transfected and CARD8-silenced cells (Fig. 4C). NLRP3 mRNA expression was reduced in unstimulated CARD8-silenced cells but was restored following CFT073 infection (Fig. 4D).

### CARD8 knockdown increases UPEC colonization and invasion of bladder epithelial cells

**Fig. 5:**
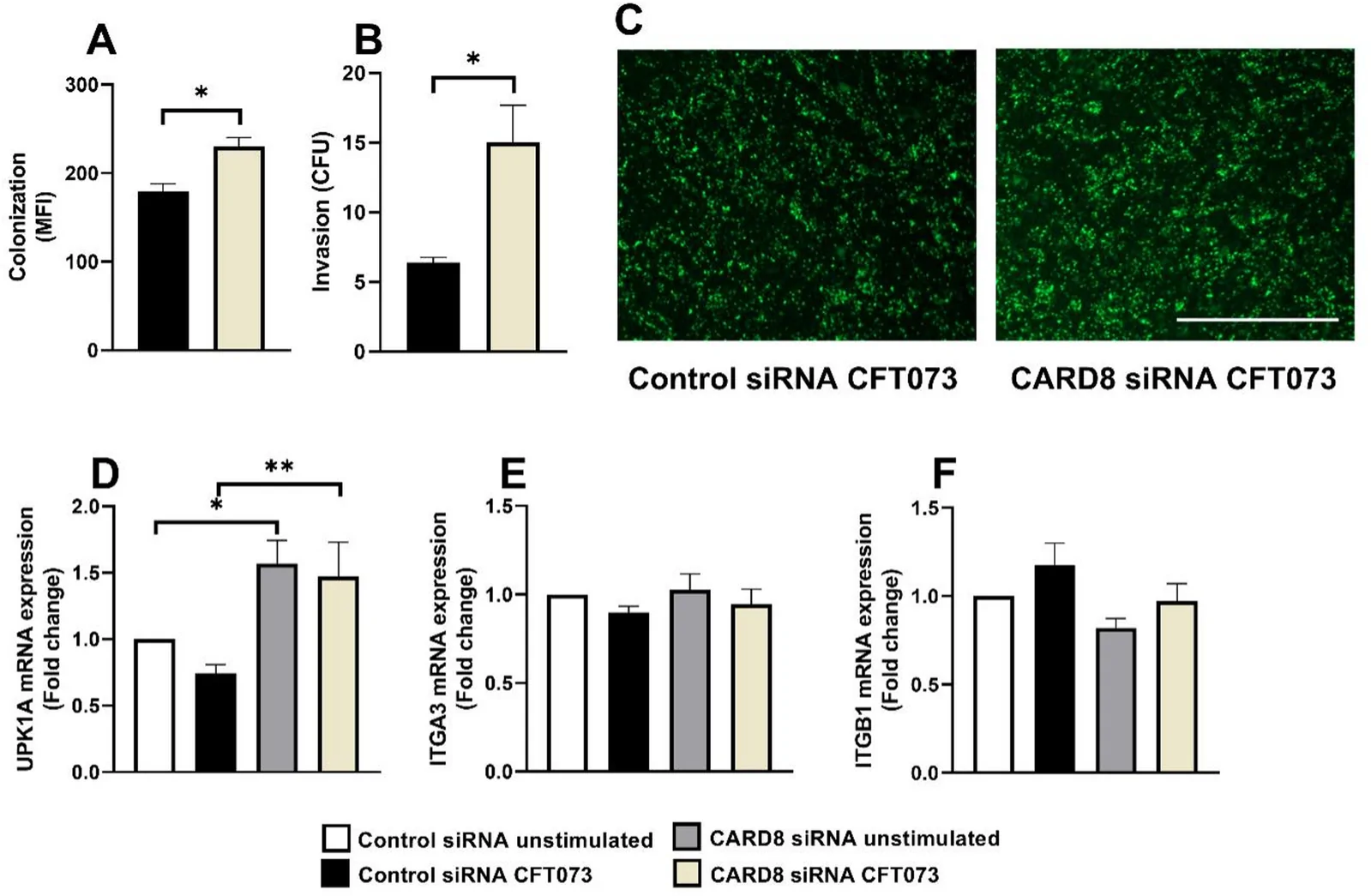
CARD8 knockdown increases CFT073 colonization and invasion of bladder epithelial cells. Colonization of HBLAK cells transfected with control siRNA or CARD8 siRNA and infected with CFT073 at MOI 100 for 3 h (**A, B**). Data is presented as mean fluorescence intensity (MFI). Invasion of HBLAK cells transfected with control siRNA or CARD8 siRNA and infected with CFT073 at MOI 10, measured by gentamicin protection assay and CFU counting (**B**). Data presented as colony forming units (CFU). Representative fluorescence microscopy images of CFT073-colonized control siRNA- and CARD8 siRNA-transfected HBLAK cells (**C**). Real-time qPCR analysis of UPK1A (**D**), ITGA3 (**E**), and ITGB1 (**F**) mRNA expression in HBLAK cells transfected with control siRNA or CARD8 siRNA and infected with CFT073 at MOI 1 for 5 h. Gene expression is presented as fold change relative to unstimulated control siRNA-transfected cells. Data are presented as mean ± SEM of *n* = 3–4 independent biological experiments. Asterisks indicate statistical significance: *p < 0.05, **p < 0.01. Scale bar: 250 μm

We next investigated the involvement of CARD8 in CFT073 adhesion and invasion of bladder epithelial cells. CARD8 knockdown significantly increased CFT073 colonization compared with control-transfected cells (Fig. 5A). In line with this, CARD8 knockdown cells also showed a significant increase in CFT073 invasion compared with control-transfected cells (Fig. 5B). Fluorescence microscopy further supported these findings (Fig. 5C). To explore whether this was associated with altered expression of epithelial receptors involved in bacterial interaction, we analyzed the mRNA expression of UPK1A, UPK3A, ITGA3 and ITGB1. CARD8 knockdown increased UPK1A mRNA expression under basal conditions compared with unstimulated control-transfected cells (Fig. 5D). In contrast, ITGA3 and ITGB1 mRNA expression were not significantly affected by CARD8 knockdown or CFT073 infection (Fig. 5E, F). UPK3A mRNA expression was not detectable in HBLAK cells under these experimental conditions. Together, these findings suggest that CARD8 contributes to limiting UPEC colonization and invasion of bladder epithelial cells possibly by downregulating the UPK1A receptor.

### HlyA modulates inflammatory signaling but not CARD8 downregulation

**Fig. 6:**
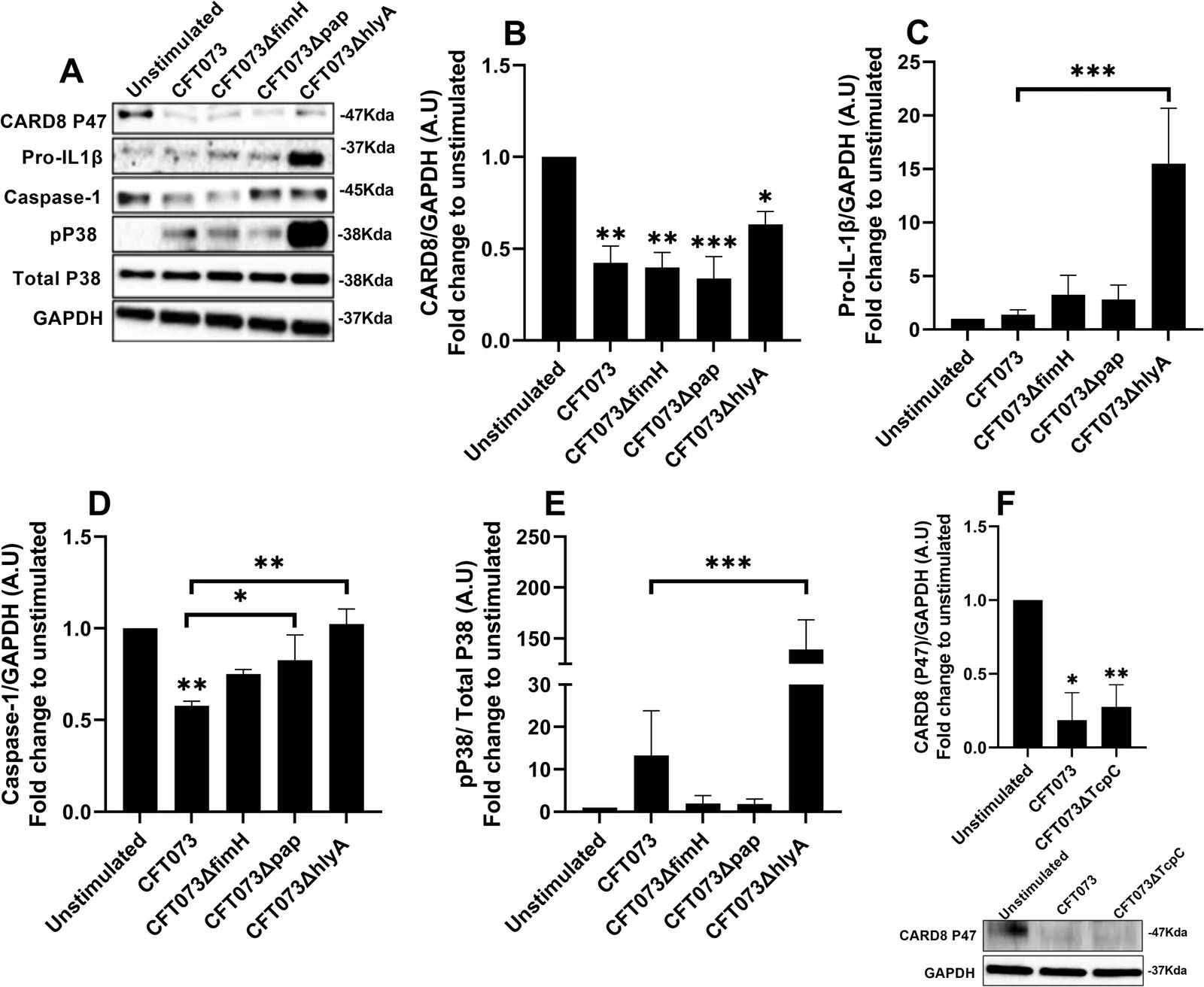
HlyA modulates inflammatory signaling. Western blot analysis of CARD8, pro-IL-1β, caspase-1, phosphorylated p38 MAPK, total p38 MAPK, and GAPDH in HBLAK cells infected with wild-type CFT073 or CFT073 mutants lacking fimH, pap, or hlyA at MOI 1 for 5 h (**A**). Densitometric analysis of CARD8 (**B**), pro-IL-1β (**C**), caspase-1 (**D**) and phosphorylated p38 MAPK (**E**). CARD8 P47 protein expression in HBLAK cells infected with wild-type CFT073 or CFT073ΔtcpC, with representative western blot and corresponding densitometric analysis (**F**). Data are presented as mean ± SEM of *n* = 3 independent biological experiments. Asterisks indicate statistical significance compared to respective unstimulated: *p < 0.05, **p < 0.01, ***p < 0.001

To investigate whether key UPEC virulence factors modulate CARD8-associated inflammatory responses, bladder epithelial cells were infected with wild-type CFT073 or CFT073 mutants lacking fimH, pap, tcpC or hlyA. Western blot analysis showed that wild-type CFT073 significantly reduced the 47-kDa CARD8 isoform compared with unstimulated cells (Fig. 6A, B). A similar reduction in CARD8 expression was also observed following infection with CFT073ΔfimH, CFT073Δpap, CFT073ΔhlyA (Fig. 6A, B) and CFT073ΔTcpC (Fig. 6F). In contrast, pro-IL-1β protein expression was significantly increased following infection with CFT073ΔhlyA compared with CFT073 infection, whereas wild-type CFT073 and the CFT073ΔfimH and CFT073Δpap mutants induced no changes (Fig. 6A, C). Caspase-1 protein expression was significantly reduced following wild-type CFT073 infection compared with unstimulated cells (Fig. 6A, D), while infection with CFT073ΔhlyA and CFT073Δpap resulted in significantly higher caspase-1 expression compared with CFT073 infection (Fig. 6A, D). Furthermore, we also found that phosphorylation of p38 MAPK was significantly increased following infection with CFT073ΔhlyA compared with CFT073 infection (Fig. 6A, E).

## Discussion

In the present study, we investigated the role of CARD8 in bladder epithelial cell responses to UPEC infection. To our knowledge, this is the first study to examine CARD8 in the context of UPEC infection in bladder epithelial cells. Previous studies have established that inflammasome-related pathways, particularly NLRP3, caspase-1, and IL-1β, are activated during UPEC infection and can influence both epithelial inflammatory responses and bacterial colonization (Demirel et al. 2018, Nagamatsu et al. 2015, Ambite et al. 2016, Waldhuber et al. 2016). However, most experimental studies of inflammasome signaling in UTI have focused on pathways that are conserved between humans and mice (Nagamatsu et al. 2015, Ambite et al. 2016, Snyder et al. 2013). CARD8 is therefore a particularly interesting and underexplored regulator, since it is not represented by a direct murine orthologue and therefore cannot be fully investigated using conventional murine UTI models (Linder et al. 2020). This limits direct in vivo validation of the physiological consequences of CARD8 regulation during UTI and highlights the importance of human-relevant models for investigating CARD8-dependent mechanisms of epithelial inflammation and host-pathogen interactions.

In this study we show that CARD8 contributes to the basal regulation of inflammasome-related and inflammatory pathways in bladder epithelial cells. In unstimulated cells, CARD8 knockdown reduced basal IL-1β and NLRP3 mRNA expression, as well as pro-IL-1β and caspase-1 protein levels. These findings indicate that CARD8 contributes to the maintenance of basal inflammatory homeostasis in bladder epithelial cells. In this context, CARD8 appears to regulate the steady-state abundance of several inflammasome-associated components rather than functioning primarily as a mediator of acute UPEC-induced inflammation. CARD8 has previously been described as a cell type- and stimulus-dependent modulator of inflammatory signaling. CARD8 has been reported to regulate inflammatory signaling through several mechanisms, including inhibition of NF-κB activation, modulation of NOD2-dependent epithelial responses, and restriction of NLRP3-dependent caspase-1 activation and IL-1β release (Bouchier-Hayes et al. 2001, Razmara et al. 2002, Kampen et al. 2010, Ito et al. 2014). In contrast, more recent studies have shown that CARD8 can also act as an inflammasome sensor, where FIIND-dependent processing enables the C-terminal UPA-CARD fragment to recruit caspase-1 and promote inflammasome activation under specific conditions (D’Osualdo et al. 2011, Sharif et al. 2021, Gong et al. 2021). Consistent with our epithelial data, Paramel et al. demonstrated that CARD8 is expressed in endothelial cells and that CARD8 positively regulate several inflammatory mediators, including IL-6, IL-8, MCP-1, CXCL1, and CXCL6 (Paramel et al. 2020). Thus, CARD8 can function either as an inflammatory regulator or as an inflammasome sensor, depending on cell type, isoform expression, and activating stimulus.

An important finding in this study was that several UPEC strains, but not a commensal *E. coli*, significantly reduced the 47-kDa CARD8 isoform in primary, spontaneously immortalized and a carcinoma bladder epithelial cell line, whereas the 60-kDa and 25-kDa CARD8 isoforms were not significantly altered. This suggests that UPEC may modulate CARD8 in an isoform-specific manner across several in vitro bladder cell lines. Since several CARD8 protein isoforms with different molecular weights have been described (Bagnall et al. 2008), the selective reduction of the 47-kDa isoform may reflect altered expression, processing, or stability of a specific CARD8 isoform during infection (D’Osualdo et al. 2011). This may be functionally relevant, as different CARD8 isoforms could have distinct roles in regulating basal epithelial homeostasis and host-pathogen interactions. However, the functional consequence of the selective reduction of the 47-kDa isoform during UPEC infection remains to be determined.

Despite the clear effects of CARD8 knockdown on the basal inflammatory state, CARD8 had a more limited role in the acute inflammatory response to CFT073. CFT073-induced IL-1β release, LDH release, and caspase-1 activity were not attenuated following CARD8 knockdown. This suggests that the acute UPEC-induced inflammasome-like response in bladder epithelial cells can occur independently of CARD8. This is consistent with previous work showing that CFT073 induces caspase-1 activation and IL-1β release from bladder epithelial cells through an NLRP3-associated pathway involving α-hemolysin, p38 MAPK, ERK1/2, ROS, and serine proteases (Demirel et al. 2018, Nagamatsu et al. 2015). Thus, CFT073 may activate sufficient CARD8-independent pathways to drive caspase-1 activity and IL-1β release even though CARD8 alters their basal levels. Taken together, these findings indicate that CARD8 influences the basal inflammatory state of bladder epithelial cells but is not required for the major acute inflammatory responses induced by CFT073 under these experimental conditions.

The IL-8 findings further illustrate the distinction between basal CARD8-dependent regulation and infection-induced inflammatory signaling. CARD8 knockdown reduced basal IL-8 release, and a similar trend was observed at the IL-8 mRNA level, although this did not reach statistical significance. Following CFT073 infection, IL-8 mRNA expression was increased. However, this transcriptional response was not reflected at the protein-release level in control-transfected cells, where CFT073 infection reduced IL-8 release. This suggests that CFT073 may uncouple IL-8 transcription from secreted IL-8 protein levels, potentially through post-transcriptional and/or post-translational mechanisms as an evasion mechanism. In CARD8-silenced cells, CFT073 infection increased IL-8 release compared with unstimulated CARD8-silenced cells. This suggests that CARD8 may influence how bladder epithelial cells regulate IL-8 protein release during infection. Previous work has shown that UPEC can suppress key epithelial cytokine responses, including IL-6 and IL-8, as part of an immune-evasion strategy (Hunstad et al. 2005, Agace et al. 1993, Ching et al. 2018, Bottek et al. 2020). Since IL-8 is regulated by inflammatory pathways such as NF-κB and MAPK signaling (Tsai et al. 2009), and since CARD8 has been linked to regulation of NF-κB- and NOD2-dependent signaling (Bouchier-Hayes et al. 2001, Razmara et al. 2002, Kampen et al. 2010), CARD8 may affect IL-8 through inflammasome-independent pathways. Together, these findings suggest that CARD8 may influence the balance between UPEC-mediated immune evasion and epithelial chemokine signaling during UTI.

Another central observation was that CARD8 knockdown significantly increased CFT073 colonization and invasion of bladder epithelial cells. This suggests that CARD8 contributes to epithelial defence mechanisms that limit UPEC adhesion or invasion. This finding is particularly interesting in relation to our previous work showing that the NLRP3 inflammasome contributes to UPEC colonization of bladder epithelial cells (Demirel et al. 2018). The opposite effects observed following CARD8 knockdown and NLRP3 deficiency indicate that CARD8 is unlikely to act simply as a component of the NLRP3 pathway. Instead, CARD8 may regulate epithelial factors that influence UPEC adhesion and invasion, such as uroplakins or α3β1 integrins (Eto et al. 2007, Zhou et al. 2001). The increased UPK1A mRNA expression, together with unchanged ITGA3 and ITGB1 expression and undetectable UPK3A expression, provides a possible mechanistic explanation, since uroplakin Ia has been identified as a urothelial receptor for type-1-fimbriated UPEC through FimH binding (Zhou et al. 2001). However, as this association is currently based on transcript-level data, further studies are required to determine whether increased UPK1A expression directly mediates the enhanced bacterial interaction observed following CARD8 knockdown. Moreover, since CFT073 itself downregulated CARD8, reduced CARD8 expression may create a more permissive epithelial environment for bacterial interaction and invasion. However, the physiological consequences of this effect remain unclear, as our in vitro model cannot determine whether CARD8 ultimately influences bacterial clearance, prolonged intracellular survival, persistence, recurrent infection, or tissue pathology. Future studies should therefore investigate whether CARD8-mediated regulation of uroplakin Ia contributes directly to UPEC entry and whether the increased invasion observed after CARD8 knockdown has consequences for subsequent infection outcome. The absence of a direct murine CARD8 orthologue also limits conventional in vivo validation, highlighting the need for more complex human-relevant models.

To determine whether major UPEC virulence factors contributed to CARD8 downregulation, bladder epithelial cells were infected with wild-type CFT073 or mutants lacking fimH, pap, tcpC or hlyA. The reduction of the 47-kDa CARD8 isoform was largely maintained across the mutants, suggesting that CARD8 downregulation is not strictly dependent on type-1 fimbriae, P-fimbriae, TIR-domain-containing protein (TcpC) or α-hemolysin alone. However, the small non-significant trend toward higher CARD8 expression after CFT073ΔhlyA infection suggests that α-hemolysin may partially contribute to this response. Previous studies, including our own, have shown that α-hemolysin has complex effects on epithelial inflammasome activation, cytokine release, and cell death (Demirel et al. 2018, Dhakal and Mulvey 2012, Nagamatsu et al. 2015). In addition, TcpC, is known to impair NLRP3 inflammasome activation (Waldhuber et al. 2016), although we did not see any effects on CARD8. The mutant experiments also showed that CFT073ΔhlyA induced higher intracellular pro-IL-1β expression and increased p38 MAPK phosphorylation, while CFT073ΔhlyA and CFT073Δpap increased caspase-1 protein expression. Since α-hemolysin can promote epithelial cell death through pyroptosis, the accumulation of intracellular pro-IL-1β after CFT073ΔhlyA infection may partly reflect reduced pyroptosis (Demirel et al. 2018, Nagamatsu et al. 2015). In parallel, the stronger p38 MAPK response after CFT073ΔhlyA infection suggests that loss of α-hemolysin removes a modulatory effect on epithelial inflammatory signaling (Demirel et al. 2018, Dhakal and Mulvey 2012, Nagamatsu et al. 2015).

These findings may also be relevant to previous genetic studies linking CARD8 variation to host responses during bacterial infection. In the context of UTI, Cheng et al. investigated polymorphisms in inflammasome-related genes, including CARD8, NLRP3 and IL1B, in pediatric patients with severe renal parenchymal infections, highlighting the potential relevance of host inflammasome genetics in severe urinary tract infection (Cheng et al. 2015). A genome-wide cell-based association study identified the CARD8 C10X polymorphism as a determinant of Salmonella-induced cell death, and functional validation showed that both the naturally occurring C10X variant and CARD8 knockdown increased caspase-1 activation and cell death (Ko et al. 2009). Our findings extend this concept to bladder epithelial cells by showing that reduced CARD8 expression not only alters inflammatory signaling but also increases UPEC colonization and invasion. Interestingly, UPEC selectively downregulated the 47-kDa CARD8 isoform, which has been proposed to escape the C10X premature stop codon. This suggests that isoform-specific regulation may be an important, but still poorly understood, aspect of CARD8-mediated antibacterial defence.

In conclusion, this study identifies CARD8 as a novel regulator of bladder epithelial responses during a UPEC infection. CARD8 contributed to basal inflammasome-related signaling, while UPEC selectively downregulated the 47-kDa CARD8 isoform. Although CARD8 was not required for acute CFT073-induced IL-1β release, LDH release, or caspase-1 activity, CARD8 knockdown altered IL-8 regulation and increased bacterial colonization and invasion, potentially through increased UPK1A expression. These findings suggest that CARD8 regulates basal epithelial immune responses and bacterial interaction during UTI.

## Data Availability

All data generated or analysed during this study are included in this manuscript.

## Abbreviations

CARD8: Caspase recruitment domain-containing protein 8
cDNA: complementary DNA
CFU: Colony-forming units
CO₂: Carbon dioxide
Ct: Cycle threshold
CXCL: C-X-C motif chemokine ligand
eGFP: enhanced green fluorescent protein
ELISA: Enzyme-linked immunosorbent assay
GAPDH: Glyceraldehyde-3-phosphate dehydrogenase
HKGS: Human Keratinocyte Growth Supplement
HUVECs: Human umbilical vein endothelial cells
IL: Interleukin
ITGA3: Integrin subunit alpha 3
ITGB1: Integrin subunit beta 1
LDH: Lactate dehydrogenase
LPS: Lipopolysaccharide
MAPK: Mitogen-activated protein kinase
MCP-1: Monocyte chemoattractant protein-1
MOI: Multiplicity of infection
NF-κB: Nuclear factor kappa B
NLRP3: NOD-like receptor family pyrin domain-containing 3
NOD2: Nucleotide-binding oligomerization domain-containing protein 2
PBS: Phosphate-buffered saline
PCR: Polymerase chain reaction
p38 MAPK: p38 mitogen-activated protein kinase
RIPA: Radioimmunoprecipitation assay
RNA: Ribonucleic acid
ROS: Reactive oxygen species
RT-qPCR: Quantitative real-time polymerase chain reaction
SEM: Standard error of the mean
siRNA: small interfering RNA
TcpC: Toll/interleukin-1 receptor domain-containing protein C
TIR: Toll/interleukin-1 receptor
TSA: Tryptic soy agar
UPEC: Uropathogenic Escherichia coli
UPK1A: Uroplakin 1A
UPK3A: Uroplakin 3A
UTI: Urinary tract infection
